# “Being kept alive—but not being supported to live”: experiences of general psychiatric inpatient care among persons with anorexia nervosa

**DOI:** 10.1186/s40337-025-01483-9

**Published:** 2025-12-03

**Authors:** Anna Sandsten, Sebastian Gabrielsson, Maria Strömbäck, Git-Marie Ejneborn Looi, Britt-Marie Lindgren

**Affiliations:** 1 Department of Nursing and Umeå Center for Gender Studies, Gender Research School, Umeå University, Umeå, Sweden; 2 Department of Health, Education and Technology, Luleå University of Technology, Luleå, Sweden; 3 Department of Community Medicine and Rehabilitation and Department of Clinical Science, Umeå University, Umeå, Sweden; 4 Department of Nursing, Umeå University, Umeå, Sweden

**Keywords:** Anorexia nervosa, Hospitalization, Diagnostic identity, Mental health, Qualitative research, Personal recovery

## Abstract

**Background:**

Although anorexia nervosa has been the focus of numerous studies, most research has been conducted within specialized eating disorder services, where the primary emphasis is on food, weight and physical markers of illness. The condition is described as difficult to treat, with persons with anorexia nervosa portrayed as being dominated by the illness and hard to engage in care. Treatment approaches are often rigid and protocol-driven, particularly in inpatient settings, where the primary aim is to preserve life. Given the limited research on psychiatric inpatient care for anorexia nervosa in settings not specialized in eating disorder treatment this qualitative study aims to explore lived experiences of being treated for anorexia nervosa in the context of general psychiatric inpatient care.

**Methods:**

The study was conducted in Sweden between August and September 2023. Eighteen women participated. Seventeen submitted written narrative texts reflecting on their experiences, nine of whom also took part in individual interviews. Only one participant agreed to being interviewed only. Data were analysed using inductive qualitative content analysis, involving a systematic abstraction and interpretation of the textual content.

**Results:**

Findings revealed one main theme, *Being kept alive—but not being supported to live*; three themes, *Being invisible as a person; Being chained by hopelessness;* and *Aiming to get on the road towards recovery;* and eight subthemes, *Lacking existential support; Having to stand up for myself; Being disconnected from real life; Being restricted; Being marked for life; Needing human connection; Finding ways to live;* and *Finding meaning and hope*.

**Conclusion:**

The findings show that persons with anorexia nervosa may experience general psychiatric inpatient care as both life-saving and unsupportive. This calls for trauma-informed, recovery-oriented care that treats lived experience as essential expertise. Future work should focus on strategies to challenge entrenched assumptions in general psychiatric inpatient care and promote approaches that respect the complexity, autonomy, and meaning making of persons living with AN.

## Abstract (plain language summary)

Anorexia nervosa is an illness that can affect a person socially, physically, and mentally. Because of the severity of the illness a person with anorexia nervosa may need inpatient psychiatric care, either in a specialized eating disorder unit or in a general psychiatric ward. Research has focused on specialized care, but persons with anorexia nervosa, especially those with other conditions such as autism, depression or self-harm are often treated in general psychiatric inpatient wards. We interviewed people with experience of such care. They said general psychiatric inpatient care could save lives, but it often failed to address underlying problems or provide hope. Many felt unseen, unheard and left to manage psychosocial aspects of the illness on their own.

## Background

Persons with anorexia nervosa (AN) experience a mental health condition with both medical and psychological needs that often requires a multidisciplinary approach [1]. Anorexia nervosa is a global disorder affecting persons across genders and age groups, although it is most frequently diagnosed in women [2]. In recent decades, incidence rates have risen among children under 15; the underlying causes remain unclear [3]. The illness is associated with markedly reduced quality of life and elevated risk of premature death due to physical complications or suicide [4]. The psychological aspects include self-criticism, low self-esteem, social isolation, feelings of entrapment, and hopelessness about the future [5].

While AN significantly impairs social functioning [6, 7], it may also serve certain psychological functions [8–10]. Rigid control over food and physical activity can provide structure, predictability, and a sense of control. For some persons, AN becomes a coping strategy to manage distressing emotions and cultivate a perceived sense of inner strength [11, 12]. However, the perspective of psychiatric inpatient care is predominantly biomedical [9, 13–16], classifying patients according to diagnostic categories and treating certain behaviours or characteristics as deviant or abnormal [14]. A treatment approach focused primarily on diagnosis and symptoms makes it difficult to understand the patient as a unique person [13]. While biomedical interventions are crucial for addressing the physical health implications of AN [1], what is often neglected in psychiatric inpatient care are the psychological, social, and emotional dimensions of the illness [9, 13, 14]. Furthermore, AN is often linked to societal ideals of femininity emphasizing thinness, self-discipline and bodily control. This may inadvertently reinforce traditional gender norms if treatment focuses solely on weight restoration rather than on the psychological dimensions of the illness [17].

Psychiatric inpatient care is provided when a person’s condition is assessed as requiring more intensive treatment than can be offered in outpatient settings [15]. In general psychiatric inpatient care (GPIC), persons with diverse diagnoses are typically accommodated in the same facility. Consequently, there is a mixture of persons with varying needs. General psychiatric inpatient care typically enforces strict regulations, with locked doors and limited personal freedom [18].

Most research on psychiatric inpatient care for AN has been conducted in specialized eating disorder units and tends to adopt a predominantly biomedical focus, emphasizing food intake, weight and other measurable parameters [14]. This perspective, while clinically important, risks overlooking the subjective, relational and contextual dimensions of care that are often central to long-term recovery and personal meaning making [14]. Furthermore, research suggests that, from the consumer’s perspective, inpatient treatment for AN often fails to adequately address patients’ psychological needs [9, 16, 19]. These insights imply that care models need to evolve beyond symptom management.

In a time when person-centred care and patients’ rights are increasingly emphasized in policy and practice, it is crucial to critically examine whether and how these ideals are realized in GPIC. While existing research has largely focused on clinical outcomes [14], this qualitative study seeks to foreground the perspectives of those who have often been spoken about, but are rarely listened to, in both research and practice. To better align care with the individual needs and preferences of persons with AN, research must take their knowledge and lived experience seriously. Their insights can offer valuable guidance on how care practices can become more responsive and supportive.

While most research on care for AN has been conducted within specialised eating disorder services, less is known about how such care is experienced in GPIC contexts. Examining experiences within this setting is essential to understand whether knowledge and assumptions derived from specialised care can be meaningfully applied in other contexts. This can best be explored by listening to patients with experiences of such treatment. Therefore, this study aims to explore lived experiences of being treated for AN in the context of GPIC.

## Methods

A qualitative design was employed, combining written narratives and individual semi-structured interviews, and findings were analysed using inductive qualitative content analysis [20, 21]. The data reporting of the study is in accordance with the Criteria for Reporting Qualitative Research (COREQ) checklist [22].

### Participants and procedures

Participants in Sweden were recruited through social media such as Facebook and Instagram, with assistance from interest groups such as Self-harm and Eating Disorder Organisation (SHEDO) and Opsynliga.se. The administrator on these mental health support pages posted information about the study’s aim and a link to a webpage containing more information and instructions on how to participate. Inclusion criteria were having own experiences of being treated for AN in GPIC and being > 15 years old. The exclusion criterion was receiving ongoing inpatient care for AN.

The study included 18 participants, all of whom identified as female and were in the age range of 19–51 years (median 28). Time since last hospitalization ranged from < 1 year to 20 (median 2.75). Two participants had experience of child and adolescent psychiatry, which in this study is classified as GPIC because of its lack of specialization in eating disorders; eleven participants had experience of adult GPIC, and five of both settings. To protect their anonymity and make it easier for them to share their story we gathered no information regarding where the participants had received inpatient care.

### Data collection

Data were collected and managed using Research Electronic Data Capture (REDCap), an electronic data capture tool hosted at Umea University in Umeå, Sweden. REDCap is a secure, web-based software platform designed to support data capture for research studies [23, 24]. The participants were asked to share their experiences of AN in the context of GPIC in written narratives in REDCap and/or participate in individual interviews. Seventeen participants contributed written texts, nine of whom also took part in interviews. The texts varied in length from 90 to 1251 (median 473) words. They were produced in response to an open prompt: “Describe how you have experienced the care you received, either in general or based on one or several specific situations. There are no requirements regarding the form of your text, your description may take the shape of a longer narrative or a shorter account”, and had a narrative character, focusing on personal experiences. In total, ten participants were interviewed, nine of whom had also submitted written narratives, and one of whom chose only to participate in an interview. The interviews were intended to enrich and expand upon the written accounts, while offering participants a choice in how to communicate their experiences.

Participants who shared a written narrative stayed anonymous, but those taking part in an interview had to provide their e-mail or telephone number to be contacted. The first author handled all contact with the participants and conducted individual semi-structured interviews during August and September 2023. Eight interviews were conducted virtually, with or without camera according to participants’ preferences, and two interviews were conducted by telephone. The interviews lasted between 45 and 96 (median 67 min). The interviews were conducted by the first author, who has extensive experience in meeting persons within psychiatric care. To ensure methodological rigour, the interview process was continuously discussed with the other authors who have substantial experience in qualitative interviewing. The first author had no prior relation to any of the participants.

If a participant had submitted a written narrative, the interview started with follow-up questions for clarification and to gain a deeper understanding of the text. An interview guide was used with open-ended questions such as: *How would you describe an ordinary day at the ward? Can you tell me about the care you were offered?* and *If you had your wish*,* how would you like GPIC to be for persons with AN?* Follow-up questions were asked to encourage participants to clarify further or develop their descriptions. All interviews were audio-recorded and transcribed verbatim by the first author. The transcripts were de-identified and saved on a USB stick stored securely in a locked safe at Umeå University.

### Analysis

Data were subjected to qualitative content analysis, which involves systematic abstraction and interpretation of the textual content [20, 21]. Transcribed interviews and the written narratives were read in their entirety several times to get a sense of the whole. The text was then divided into meaning units, which were condensed and labelled with codes in line with the study’s aim. The codes were sorted into groups based on similarities and differences. Groups of codes were abstracted, interpreted and grouped into subthemes. For example, the codes “new rules”, “missing out”, “being displaced” and “fading joys” were grouped, abstracted and interpreted to form the subtheme “Being disconnected from real life”. Another example of codes grouped were “looking at life in new ways”, “rising from meaningful relationships” and “being able to live with AN” which ended up as the formulated subtheme “Finding ways to live”. Subthemes with similar content were grouped together and abstracted and interpreted into broader themes. In the final step one main theme was formulated (see below).

### Findings

The participants’ lived experiences of being treated for AN in the context of GPIC are presented as one main theme, *Being kept alive—but not being supported to live*; three themes, *Being invisible as a person; Being chained by hopelessness;* and *Aiming to get on the road towards recovery*; and eight subthemes, *Lacking existential support; Having to stand up for myself; Being disconnected to real life; Being restricted; Being marked for life; Needing human connection; Finding ways to live;* and *Finding meaning and hope* (Table 1). In the following, the main theme, themes and subthemes are presented as interpretative narratives supported by several illustrative quotations from different participants:

**Table 1:** Overview of the main theme, themes and subthemes revealed in the analysis

Main theme	Themes	Subthemes
Being kept alive—but not being supported to live	Being invisible as a person	Lacking existential support
		Having to stand up for myself
		Being disconnected from real life
	Being chained by hopelessness	Being restricted
		Being marked for life
		Needing human connection
	Aiming to get on the road towards recovery	Finding ways to live
		Finding meaning and hope

### Being kept alive—but not being supported to live

The main theme reflects participants’ overall experiences of GPIC which was described as solely keeping them alive but not supporting them in living. Rather than being seen as persons, they felt they were objectified as bodies under control, where food, weight and routines dominated everyday life. Dialogue, personal adaptation, and participation were largely absent, making the participants feel invisible. Care was rarely experienced as fostering meaning, relationships or recognition of one’s own voice. Instead, admission into general psychiatric inpatient care often evoked a sense of being chained by hopelessness. Care was experienced as focusing on meeting discharge requirements rather than facilitating genuine change. The participants described longing for a different kind of care grounded in understanding, collaboration and respect for their own narrative. They emphasized that, despite the limitations of care, their aim was getting on the road towards recovery.

#### Being invisible as a person

Participants’ experience of being invisible as a person to GPIC staff, was characterized by staff’s one-sided focus on physical symptoms of AN, medical stabilization and standardized routines, while the participants’ personal narratives, psychological needs and subjective suffering were rarely considered in care. Being invisible as a person was described as lacking existential support, often having to stand up for oneself against staff, and feeling increasingly disconnected from real life. Together, these experiences contributed to a pervasive sense among participants of invisibility in GPIC, which involved feeling that they were not recognized as unique persons and they were distanced from meaningful engagement in everyday life.

##### Lacking existential support

Existential support, understood as a process of working on inner change, attending to one’s emotional state, and engaging in personal meaning-making, was lacking in the participants’ experiences. Instead, care was described as being guided by an implicit understanding of the diagnosis as objective and unambiguous. The participants felt misunderstood when they perceived that staff’s actions were based on assumptions about “what AN is” rather than willingness to find out how the illness was expressed by them as persons. The participants described how a diagnostic framework often replaced genuine listening. They experienced care as a process in which they had to follow rules, accept treatment and demonstrate obedience.

To me, the eating disorder was, and still is, a symptom, but no one there [at the ward] cared about that. No one asked how I felt. I was pushed to gain weight, into a body I wasn’t used to, back into the emotions I had suppressed through starvation. No one asked how that felt. (Participant 20, written narrative)

When attempting to articulate feelings of anxiety, trauma, identity struggles or psychological conflict, the participants rarely encountered interest or therapeutic engagement. They reported that they were not believed when expressing psychological suffering. Instead, their body, with its measurable parameters, became the primary focus, while subjective experiences were ignored.

I definitely experienced the consequences that a lack of therapeutic alliance can have on treatment. As I said, if you’re wondering whether I experienced the care as hopeful… I really didn’t. I was even told by a doctor, “We don’t know what to do with you.” That wasn’t exactly hopeful or encouraging for someone already feeling broken, like a failure, and fundamentally damaged. (Participant 2, interview)

The perceived lack of psychological support at times meant that the participants felt compelled to “act sicker [sic]”, for example by maintaining a low weight or engaging in self-harming behaviours, in order for their suffering to be noticed and acknowledged. At the same time they described feelings of failure and persistent fear of receiving less support once their bodies no longer signalled acute risk.

It feels like, if my weight isn’t extremely low, they won’t give me the same support. But in my head, I might be struggling even more now than when I was at my lowest weight. That makes me feel like a failure for not being thin enough. Like I’m exaggerating, or just not good at being sick—like, I’m doing it wrong. And that kind of thinking gets in the way of trying to get better. (Participant 8, interview)

##### Having to stand up for myself

The participants did not perceive care as supportive, but rather as something to endure or defend themselves against. Their body became a site of power struggle and resistance in the form of silence, refusal or breakdown, which often became the only possible mode of expression when words were insufficient. The participants described developing strategies such as refusing food or committing self-harm, even when these were self-damaging. For some, this became a way to assert their voice in a context where other forms of agency were unavailable.

To go to war, that’s really how it felt. “War” became a way to describe the experience of being cared for, whether it was voluntarily or not. It [“war”] also captures what daily life looked like: a constant battle, primarily against myself, but also against the systems I was part of. (Participant 7, interview)

Rather than alleviating suffering, there was a risk that care actually deepened the suffering, as the internal battle the participants faced was replaced by a struggle against the institution and its representatives. The participants experienced that if they questioned care in GPIC or expressed fear and frustration they risked being dismissed by staff as “manipulative” or “difficult”.

The staff categorize and judge all persons with underweight in the same way, and I felt that wasn’t me. I wanted them to treat me as a person, not as a disease. (Participant 13, interview)

##### Being disconnected from real life

The participants described how the lives they were living or expected to return to after their time in GPIC were not incorporated into the care provided. They described the fear that meaningful activities or experiences that brought or had previously brought them joy would be withdrawn as punishment, for example if weekly weight gain was insufficient. This reinforced the participants’ perception of not being recognized as the persons they were, but rather, of being treated primarily as a diagnosis.

There’s been very little ambition or opportunity for me to develop a healthy relationship with my wellbeing, body or food. Instead, it’s all been about enduring a confined hospital environment filled with restrictions, rules, prohibitions, inactivity, uncertainty and relative loneliness—a system based on punishment rather than encouragement. (Participant 4, written narrative)

Moreover, the participants experienced a lack of continuity and preparation for the life that awaited them after discharge, which they felt, reinforced a sense of being disconnected from the outside world. The care was experienced as compounded by structurally passive and monotonous daily routines. Meals, rest and supervision structured the days, while elements of meaningful activity, engagement or preparation for life beyond the ward were described as either absent or arbitrary.

#### Being chained by hopelessness

The participants described that the care provided did not improve their psychological wellbeing, which lead to feelings of hopelessness and mistrust. They experienced restrictions, felt marked for life by their time in GPIC, and emphasized a persistent need for human connection.

##### Being restricted

The participants experienced the care environment as a space where their bodies became battleground for control and a place for coercion and struggle. Rather than offering safety and recovery, GPIC was shaped by rules, surveillance and interventions that systematically restricted the participants’ autonomy. Decisions concerning the most basic aspects of daily life, such as using the bathroom, showering or eating, were removed from the participants’ control and subordinated to staff assessments and routines. This generated a profound sense of powerlessness, where the participants’ will and integrity were subordinated to the institutional logic of the system.

I felt very vulnerable and helpless. I had no control over what was happening to me, others made those decisions for me. I had no privacy at all; they were with me even in the shower. (Participant 8, interview)

Several participants described the therapeutic relationship as a struggle for power, where any deviation from expected behaviour could result in punitive measures. Refusing to eat, sitting on the floor, or expressing distress were interpreted as defiance, and were often met with coercion and threats rather than curiosity or understanding. Hence, the participants perceived staff as lacking understanding and knowledge regarding their eating disorder.

Every day was a constant negotiation, how to avoid tube feeding, how to avoid eating at all. The days were filled with crises, broken promises and a lack of trust in staff who knew little about eating disorders or anorexia. They would say, “If you manage to eat this today, you won’t need the feeding tube”, which sounded reassuring. But when a nurse or caregiver misinterpreted the doctors’ instructions or promised things they shouldn’t promise, the day would still end with me not meeting the requirements and being tube-fed anyway. (Participant 7, interview)

Furthermore, the participants described a definite lack of knowledge among staff about neurodevelopmental conditions such as autism, which could further exacerbate a care situation.

I don’t think they understood my need for predictability, like giving me a heads up before coming in to make my bed, take a blood sample or replace my feeding tube. When they didn’t warn me that a medical student or an extra nurse would be present, I got extremely stressed and panicked. If they didn’t recognize that, I would end up hurting myself. It felt like they didn’t see how this was linked to my autism, even though it was obvious. And in the end, their response was often to restrain me; I spent much of my time [there] in belts. (Participant 15, interview)

##### Being marked for life

Coercive measures such as physical restraint, tube feeding and mechanical belt restraints were described by the participants as traumatizing. They described that coercive measures were often applied routinely rather than as a last resort. At the same time, the participants felt that there was a lack of recognition of how staff behaviour itself could provoke anxiety or panic, thereby escalating the likelihood of conflict and coercion.

I was subjected to many forced feedings under restraint, which deeply traumatized me. Being held down as a teenager by up to five large adult caregivers is a feeling that will stay with me forever. Some staff didn’t seem to know what to do, they shouted at me, and I shouted back, struggling to break free. Their stress fed my anxiety, and I felt like they had no control over the situation. When they let go, I ran into the corridor, screaming and crying in a way I didn’t know was possible—out of pure fear. For a long time after, I couldn’t bear to have staff near me. (Participant 18, written narrative)

By contrast, some participants described coercion as a paradoxical relief, a temporary suspension of responsibility. Overall, however, these experiences were marked by trauma and a loss of trust. The participants described how feelings of isolation and guilt were further intensified when staff lacked emotional presence and understanding or did not take the time to listen. Comments from staff emphasizing how distressing forced interventions were for them, such as some staff members made during tube feeding, amplified the participants’ sense of being a burden, which in turn reinforced a destructive self-image.

This might be contradictory, but in a way it was a relief when staff threatened me with tube feeding. […] You constantly have that inner voice telling you to escape, to lie, to manipulate, to hide as much food as possible. But when staff brings in their restraint team, it becomes impossible for me to win that inner battle, and in that moment, I can let it go. (Participant 7, interview)

I can’t ask for help when I have anxiety; I completely freeze. I believe it’s connected to my PTSD [post-traumatic stress disorder]; it becomes impossible, and I shut down. Most of the time, I’m unable to speak; it’s a total block. And if you can’t communicate verbally, it becomes a problem because you’re seen as uncooperative. (Participant 14, interview)

Furthermore, the participants perceived a lack of understanding among staff, particularly concerning the complexity of eating disorders. This fostered mistrust among the participants towards the care provided, as well as feelings of hopelessness. Experiences of being disbelieved, misunderstood or stripped of autonomy often led to long-term avoidance of health care, even when they needed support.

I have the health care system to thank for the fact that I’m alive, but the care I received has also left me with lifelong scars. (Participant 20, written narrative)

##### Needing human connection

The participants described the profound significance of relationships with some staff they had encountered, emphasizing that these connections were vital for their survival and for sustaining their efforts towards recovery. They highlighted the importance of meeting someone who truly saw them, who remained present in moments of difficulty, and who invested time in building a relationship.

What I’ve missed is staff spending time with me outside of just the forced measures. You’re left alone with your anxiety, except when it’s time for tube feeding - that’s the only human contact you get. And it really affected the way I saw myself, in a very negative way. (Participant 8, interview)

The participants perceived care more as a space of correction than as one of healing. They described a sense of belonging when staff saw them as unique persons, although genuine relationships with staff were the exception. Furthermore, they experienced the quality of care as inconsistent and largely dependent on individual staff members rather than on coherent structures and treatment models.

What I felt, looking back is that I felt very monitored. It wasn’t exactly … a punishment, but it felt like … well, someone was sitting with me just to make sure I wouldn’t vomit. […] I think if the staff had said something like, “We’re sitting with you because we know it’s common to feel anxiety after meals, and we don’t want you to be alone with that”, then I think it would have felt much better. (Participant 6, interview)

According to the participants, there was an absence of opportunities for reflection, dialogue or meaning-making relationships, which deepened the experience of being invisible as a person. The participants perceived that the care environment was a state to be endured, a place where they were objectified as a body that could be corrected rather than a human being who was to be understood.

I needed to talk, to receive support, and sometimes just a hug. I needed to feel valuable as a person, not like something the cat dragged in. (Participant 13, interview)

#### Aiming to get on the road towards recovery

The participants described how they grappled with the often painful realization that the help they received in GPIC rarely aligned with their actual needs. At the same time, they reported that certain experiences, relationships, and human touch could open up space for agency, enabling them to find new ways in life and cultivate meaning and hope, thus supporting their journey towards a life where AN was not affecting everything.

##### Finding ways to live

Getting well was not always portrayed as freedom from illness, but rather as the capacity to live with the illness, and despite it. This often involved mourning the life one did not get to live, while simultaneously creating new, adapted dreams.

I don’t have a family, I can’t have kids, I can’t have a partner because no one could stand living with me in my starvation. And that’s fine, I’ve accepted that. But I can still have other kinds of relationships, and I can still do things - even if it’s in a completely different way than a free person with a free relationship with food could. (Participant 7, interview)

Despite some positive experiences, the participants described that progress came not as an outcome of care, but often as an outcome of resistance to it. While care was described as lifesaving in an acute phase, it was rarely seen as supportive in the long term. Instead, the participants emphasized the importance of finding meaning elsewhere: through work, relationships, volunteering or participation in others’ journeys of getting well.

I’ve sought help elsewhere […]. I’ve been able to talk about things other than food and weight with someone who actually saw me as a person, and I’ve found more meaning in my life. Now I’m more stable, not “recovered” by any means, but nowhere near as sick as I used to be. (Participant 20, written narrative)

##### Finding meaning and hope

The participants often felt compelled to adapt to a logic of care that prioritized routines and manuals over personal needs. At the same time, certain staff members were able to offer a sense of human presence, trust and connection through small yet meaningful acts. When such encounters occurred, they were described as crucial for the participants’ capacity to endure and move forward. Individualized and flexible approaches, in which participants felt seen and validated, were described as enabling small but significant steps towards getting better.

It wasn’t until my sixth admission for anorexia that a doctor finally realized the standardized treatment everyone received wasn’t working for me - obviously, since I kept coming back in a worse and worse condition. That’s when they decided to give me an individualized meal plan, where, for example, I wasn’t forced to drink milk or juice, things I had never consumed before in my life. And as a result I could avoid being tube-fed. (Participant 18, written narrative)

The participants described the therapeutic relationship as a potential resource - but only when it was built on trust, responsiveness and recognition of their own narrative and needs. When such a relationship emerged, the care became part of a meaningful process of change; in its absence, however, the risk of further harm and increased resistance to future care was significant.

I remember one staff member who was sitting in meal support with me; she didn’t work there very often, but she’d picked up on the fact that I like horses. So when I was sitting there crying, trying to eat, she started talking about horses. With that small gesture, she made me feel seen. I could tell, even at the time, that she brought it up intentionally to make things easier for me, and it actually did help. (Participant 6, interview)

## Discussion

The findings describing the lived experiences of being treated for AN in GPIC bring to light a central paradox: persons who are severely ill and actively seek help may find themselves in a context where their autonomy is reduced and their control diminished, and where they feel invisible and misunderstood. Therefore, at the very moment when they most need support, they encounter a context they experience as unsafe and unhelpful, yet with no alternative avenues of care available. Although GPIC was described by some as life-saving, and certain staff interactions were described as deeply meaningful, the overall experience was more often characterized by a sense of being kept alive rather than being supported to live—an experience that provided limited psychological benefit, and was marked by persistent misrecognition and, in some cases, trauma. The participants described this misrecognition as stemming from a mismatch between the focus of treatment and how they themselves understood AN: while they experienced treatment as primarily aiming to stabilize measurable factors such as weight and food intake, they emphasized that, for them, AN functioned as a strategy for regulating overwhelming emotions, often grounded in earlier life experiences. Despite this, the participants expressed a wish to get on the road towards recovery and a meaningful life, something they often had to pursue largely outside the care they received.

To deepen the understanding of the findings, they are here discussed through the lens of three theoretical perspectives: first, the *biomedical perspective* of care provides a starting point for considering how practices can become centred on survival rather than on living. Second, the conception of *epistemic injustice* can help analyse the potential silencing of a person’s voice, while, thirdly, *recovery-oriented perspectives* highlight dimensions of care that were perceived as missing. In addition, concepts from *trauma-informed care* are drawn upon to suggest how treatment approaches that overlook past adversity might inadvertently reinforce suffering rather than alleviate it.

The *biomedical perspective* sees disease as a localized biological deviation. Health care is organized around identifying and correcting such deviations [25]. This perspective builds on Foucault’s [26] concept of the “medical gaze”, a clinical perspective where patients are treated as objects of observation, separate from their subjective experiences. In our findings participants described treatment as being organized primarily around weight, food intake and observable behaviours. While they recognized the importance of focus on bodily survival, they also experienced it as leaving little space for personalized care, dialogue or personal meaning. When standard treatment protocols proved ineffective, care often became rigid and impersonal, with few or no alternative strategies offered. In such situations, the participants felt that they were perceived, not as persons in need of tailored support, but as difficult, unmotivated or resistant—a perception reinforced by clinical perspectives suggesting that some persons simply cannot be helped because of the severity of the diagnosis.

This may reflect a broader biomedical logic that prioritizes bodily survival over the complexity of lived experience in psychiatric inpatient care for AN [16]. Studies spanning nearly two decades point to a striking continuity in how patients experience such care: their perspectives are frequently disregarded or pathologized, with their expressions of distress interpreted not as legitimate concerns but as symptoms of the illness [13, 16, 27, 28]. Furthermore, persons with AN have described how the stereotypes of being “just another anorexic” is projected onto them, diminishing their credibility as knowers of their own experience [13, 16, 28]. Previous research conducted within specialized eating disorder inpatient units has identified similar tensions between medical stabilization and psychological recovery [16, 29]. The similarities between patients’ experiences in GPIC and specialized eating disorder units indicate that it is not primarily the context that shapes these experiences, but how treatment is organized and enacted by staff. Taken together, these accounts illustrate how the biomedical framework, while striving for physical survival, can simultaneously undermine the voices of persons with AN.

Fricker’s [30] concept of *epistemic injustice* provides a useful lens for understanding how persons with AN may be systematically discredited or misunderstood in GPIC. “Epistemic injustice” refers to forms of injustice related to knowledge, where personal experiences are undervalued or rendered unintelligible. This occurs either when testimonies are not taken seriously (testimonial injustice) or when a person lacks access to the conceptual resources required to render their experiences intelligible in the dominant interpretive framework (hermeneutical injustice). Testimonial injustice was evident in the participants’ accounts of being perceived as manipulative or deceitful, when their expressions of distress were dismissed as symptoms rather than recognized as meaningful. Such entrenched stereotypes about AN foster mistrust of the person’s voice, thereby undermining their capacity to shape their own care. Hermeneutical injustice emerged in the GPIC staff’s failure to adapt treatment to neurodivergence, such as autism. The participants described environments lacking both basic accommodation for sensory sensitivities, and predictable routines. This heightened the participants’ anxiety, which often led to further misinterpretation of their needs. Epistemic harms make personal experiences difficult to express and may further be difficult for staff to interpret within the prevailing biomedical frameworks.

The study’s findings therefore highlight that persons with AN in GPIC may have to engage with a care system that is life-saving but at the same time epistemically unfair. According to Isobel [31], trauma-informed practices offer an important counterpoint. Rather than interpreting distress as pathology or resistance, trauma-informed approaches seek to understand behaviours considering past harm, and emphasizing safety, choice, collaboration, trust and empowerment. From an epistemic perspective, such approaches create conditions where a person’s testimony may be heard and validated, reducing the risk of testimonial injustice, while also expanding the interpretive resources needed to address hermeneutical gaps [30]. The present findings may exemplify that, without trauma-informed practices, there is a risk of care remaining narrowly corrective, leaving little room for frameworks that acknowledge a person as a credible agent in their own process of change.

*Recovery-oriented* care is a framework that challenges traditional, hierarchical models of psychiatric practices which marginalize the voices of persons in care in favour of clinical authority [32]. The participants expressed a desire for GPIC that would place greater emphasis on them as persons, both in the present moment and in relation to their broader life context. They described improved well-being as encompassing not only life before and after discharge, but also engagement in personal interests, self-understanding, and the development of new ways of relating to life. They described having to find meaning and manage their well-being largely independently of formal care. However, they also highlighted the significant and sometimes decisive positive impact of supportive staff, noting that trusting, empathic relationships could substantially enhance the experience of care. These accounts suggest that trauma-informed approaches that prioritize safety, choice, collaboration, trust and empowerment [31] can be particularly important for addressing epistemic injustices in care settings. By recognizing persons in care as credible sources of knowledge and active participants in shaping their own pathways towards recovery, such approaches not only validate lived experiences, even when they challenge biomedical norms, but also create conditions in which care can be genuinely responsive to personal needs [33, 34].

The findings highlight the need for recovery-oriented approaches to improve clinical practice. This includes validating a person’s own understanding of their illness, engaging in genuine dialogue, and offering care that acknowledges the complexity of each person’s situation. It also includes supporting connections to life outside the ward, ensuring access to meaningful daily activities and fostering trusting relationships with staff, with nurses often uniquely positioned to take on these roles [35].

According to Fricker [30], there is a risk of epistemic injustice when patients’ voices are neglected in care, or when a person is framed as “resistant” or “manipulative” rather than as a credible narrator of their own experiences. Isobel and Edwards [36] point out how trauma-informed approaches offer one way forward, emphasizing safety, predictability and collaboration, and avoiding of re-traumatization. In clinical practice, this can mean creating ward environments that are structured but not punitive, involving persons in care in treatment decisions, and validating their accounts of distress rather than interpreting them solely as symptoms of illness. Recovery-oriented perspectives further highlight the need to recognize the person’s own goals and understanding of their illness. Nurses close everyday contact with persons in care enables them to attend to the emotional and relational aspects of care [35]. Hence, through consistent communication, a genuine interest in the person beyond the diagnosis, and supporting connections to everyday life, nurses can foster trust, instil hope, and strengthen long-term recovery for persons with AN. As demonstrated by Salberg et al. [37], structured recovery-oriented programmes led by nurses in inpatient settings can provide a concrete framework for such practices, showing the potential for nursing to actively shape a more personalized and recovery-oriented care.

### Strengths and limitations

While this study provides valuable insights into how persons with AN experience GPIC, some limitations must be acknowledged. First, regarding the variation of depth and richness in the data, although 18 persons participated, not all were interviewed, and some of the written narratives were less rich in content. Nevertheless, the interviews offered detailed accounts that, when combined with the written material, illuminate a paradox between the structure and prevailing practices of GPIC and the lived experiences of persons with AN. Second, the transferability of the study may be limited as all participants were Swedish-speaking females with no gender or ethnic diversity, factors warranting consideration when interpreting these findings. Third, the use of social media for recruitment may have resulted in some persons missing the opportunity to participate, which may have influenced the diversity and scope of experiences captured. Moreover, recruiting through online platforms and websites directed at AN (e.g., SHEDO, Opsynliga.se) likely introduced a selection bias, as it may have attracted participants who are more engaged in online communities or more willing to share their experiences publicly. Finally, this study had no researchers with lived or living experience of AN directly involved in the research process. Involving such researchers would likely have strengthened the study and provided opportunities for co-produced or co-designed research. To partly address this limitation, we sought to incorporate lived experience perspectives indirectly by drawing on previous work conducted by researchers with lived or living experience, such as O’Connell [13], Deegan [33] and Elwyn [27]. This approach aimed to position experiential knowledge as a central component of the conceptual framework and to acknowledge existing hierarchies of knowledge in the field. Future research should prioritise the inclusion of lived experience researchers to further enhance the depth, relevance, and legitimacy of inquiry in this area. This study’s findings should be interpreted with caution when applied to broader populations.

## Conclusion

This study offers insights into how some persons with AN perceive GPIC as both life-saving and unsupportive. The findings highlight the need to integrate trauma-informed and recovery-oriented approaches that value lived experience as essential knowledge. Embedding this expertise into care planning, service delivery and research is crucial for avoiding tokenism and fostering responsive treatment. The challenge is to create systems where the perspective of persons with AN are not merely heard but are recognized as indispensable drivers of change. Future work should focus on strategies to challenge entrenched assumptions within GPIC and promote approaches that respect the complexity, autonomy and meaning making of persons living with AN.

## Data Availability

The datasets generated and analysed during the current study are not publicly available owing to privacy and confidentiality concerns. Sharing these qualitative data could compromise the participants’ anonymity, as the data relate to personal experiences of psychiatric care. Data may be available from the corresponding author on reasonable request, subject to approval by the relevant ethics committee.

## Abbreviations

AN: Anorexia nervosa
COREQ: Criteria for Reporting Qualitative Research
GPIC: General psychiatric inpatient care
PTSD: Post-traumatic stress disorder
REDCap: Research Electronic Data Capture
SHEDO: Self Harm and Eating Disorders Organisation
